# High Dietary Magnesium Intake Is Associated with Low Insulin Resistance in the Newfoundland Population

**DOI:** 10.1371/journal.pone.0058278

**Published:** 2013-03-05

**Authors:** Farrell Cahill, Mariam Shahidi, Jennifer Shea, Danny Wadden, Wayne Gulliver, Edward Randell, Sudesh Vasdev, Guang Sun

**Affiliations:** 1 Division of Medicine, Faculty of Medicine, Memorial University of Newfoundland, St. John’s, Canada; 2 Discipline of Laboratory Medicine, Faculty of Medicine, Memorial University of Newfoundland, St. John’s, Canada; Brigham & Women’s Hospital, and Harvard Medical School, United States of America

## Abstract

**Background:**

Magnesium plays a role in glucose and insulin homeostasis and evidence suggests that magnesium intake is associated with insulin resistance (IR). However, data is inconsistent and most studies have not adequately controlled for critical confounding factors.

**Objective:**

The study investigated the association between magnesium intake and IR in normal-weight (NW), overweight (OW) and obese (OB) along with pre- and post- menopausal women.

**Design:**

A total of 2295 subjects (590 men and 1705 women) were recruited from the CODING study. Dietary magnesium intake was computed from the Willett Food Frequency Questionnaire (FFQ). Adiposity (NW, OW and OB) was classified by body fat percentage (%BF) measured by Dual-energy X-ray absorptiometry according to the Bray criteria. Multiple regression analyses were used to test adiposity-specific associations of dietary magnesium intake on insulin resistance adjusting for caloric intake, physical activity, medication use and menopausal status.

**Results:**

Subjects with the highest intakes of dietary magnesium had the lowest levels of circulating insulin, HOMA-IR, and HOMA-ß and subjects with the lowest intake of dietary magnesium had the highest levels of these measures, suggesting a dose effect. Multiple regression analysis revealed a strong inverse association between dietary magnesium with IR. In addition, adiposity and menopausal status were found to be critical factors revealing that the association between dietary magnesium and IR was stronger in OW and OB along with Pre-menopausal women.

**Conclusion:**

The results of this study indicate that higher dietary magnesium intake is strongly associated with the attenuation of insulin resistance and is more beneficial for overweight and obese individuals in the general population and pre-menopausal women. Moreover, the inverse correlation between insulin resistance and dietary magnesium intake is stronger when adjusting for %BF than BMI.

## Introduction

Type 2 diabetes comprises 90% of all diabetic cases and has become an ever increasing healthcare challenge as the number of people affected reaches epidemic proportions [1]. The prevalence of this condition is expected to reach over 438 million people globally by the year 2030 and carries with it a significant fiscal burden [2]. This is especially relevant to the Canadian province of Newfoundland and Labrador considering it has one the highest rates of diabetes and obesity in Canada. Currently, 9% of the population of Newfoundland and Labrador struggle with diabetes which corresponds to an annual cost of $254 million dollars [3]. The Canadian Diabetes Association has projected that by the year 2020 over 15% of Newfoundland and Labradorean’s will be diabetic with an annual health care cost exceeding $360 million dollars [3]. Although there is currently no medical intervention capable of preventing the development of diabetes, simple lifestyle modifications (such as increased physical activity, moderate weight loss, and eating behavior modifications) have been shown to attenuate the onset of type 2 diabetes [1], [4]. However few studies to date have investigated the association between dietary micronutrient intake and insulin function in the general population.

Magnesium, a cofactor required in over 300 enzymatic reactions, is the fourth most abundant cation in the human body involved with both glucose metabolism and insulin homeostasis [5], [6]. Although recent evidence has suggested that dietary magnesium intake may play an important role in enhancing insulin sensitivity, population based studies have found conflicting evidence regarding the potential benefit of dietary magnesium intake. Several studies have correlated low dietary magnesium intake [7]–[9] and serum magnesium [10] with increased insulin resistance [11]–[13]. However, other studies do not support the proposed protective effect that dietary magnesium can attenuate the development of diabetes [14]–[16]. Of note, most studies have not adequately controlled for adiposity in their analysis. Instead of controlling for relative body fat, the majority of studies have utilized the body mass index (BMI) which cannot accurately distinguish fat mass from and fat-free mass [17]. Since adiposity is the parameter most closely linked with the development of insulin resistance [18], controlling for this important confounding factor is critical. Therefore we designed the present study to investigate the association between magnesium intake and insulin resistance in the Canadian province of Newfoundland and Labrador, taking into consideration age, gender, caloric intake, physical activity, medication use, smoking status, menopause and adiposity.

## Methods

### Ethics Statement

Ethics approval was obtained from the Human Investigation Committee, Faculty of Medicine, Memorial University, St. John’s, Newfoundland, Canada. All subjects provided written and informed consent before participation in this study.

### Subjects

All 2330 subjects (600 men 1725 women) from the current study are volunteers from the general population of Newfoundland and Labrador and the cohort for our ongoing CODING (Complex Diseases in the Newfoundland Population: Environment and Genetics) Study. The CODING study is a large-scale nutrigenomics investigation [17], [19], [20]. Eligibility of participants for the CODING study are based upon the following inclusion criteria: 1) ≥19 yrs of age; 2) at least a third generation Newfoundlander; and 3) healthy, without any serious metabolic, cardiovascular, or endocrine diseases. The primary method of subject recruitment for the CODING study was the use of posters and handouts. This literature was distributed throughout public facilities (offices, hospitals, and gyms) in the city of St. John’s, Newfoundland and Labrador. All subjects completed screening questionnaires providing information about physical characteristics, physical activity, health status, and dietary practices. Anthropometrics, body composition, and biochemical measurements were collected following a 12 hour fast.

### Physical Activity

Physical activity patterns were measured using the ARIC-Baecke Questionnaire, which consists of a Work Index, Sports Index, and Leisure Time Activity Index [21].

### Dietary Magnesium Assessment

Dietary intake patterns of each participant were assessed using a 124 item semi-quantitative Willett Food Frequency Questionnaire (FFQ) [22]. The Willett FFQ is one of the most commonly used dietary questionnaires for large-scale epidemiologic studies [23]. The Willett FFQ obtains from subjects, the number of weekly servings consumed of specific food item(s). NutriBase Clinical Nutrition Manager (version 8.2.0; CybersoftInc, Phoenix, AZ) software package was used to convert weekly serving values into mean daily serving values to calculate the total daily intake of magnesium (mg/day) for each individual. The nutritional information, including dietary magnesium intake, was computed for all subjects in the CODING study [19].

### Anthropometric and Body Composition Measurements

Subjects were weighed (Health O Meter, Bridgeview, IL) to the nearest 0.1 kg in standardized clothing (hospital gown). Height was measured using a fixed stadiometer (nearest 0.1 cm). Body mass index (kg/m^2^) was calculated as weight in kilograms divided by participants’ height in meters squared. Whole body composition measurements including fat mass, lean body mass, and bone mineral densities were measured using dual-energy X-ray absorptiometry (DXA) Lunar Prodigy (GE Medical Systems, Madison, WI). DXA can produce an accurate measurement of adipose tissue within the body with a low margin of error. For this reason, DXA is considered to be one of the most accurate measurements of adiposity. DXA measurements were performed on subjects following the removal of all metal accessories, while lying in a supine position as previously described by us [17], [19], [20]. Body fat percentage (%BF) is determined as a ratio of fat mass to total body mass (Version 12.2 of the enCORE software). Quality assurance was performed on our DXA scanner daily and the typical CV was 1.3% during the study period.

### Biochemical Measurements

Serum concentrations of glucose and magnesium were measured on an Lx20 analyzer (Beckman Coulter Inc., Fullerton, CA) using Synchron reagents. Serum insulin was measured on an Immulite Immunoassay analyzer. Insulin resistance and beta cell function were determined with the homeostasis model assessment (HOMA), as described by Matthews et al [24].
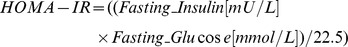


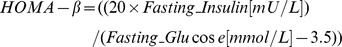



### Smoking, Medication and Menopausal Status

A self-administered screening questionnaire was used to collect information about the subjects’ demographics, personal and family medical history, medication use (yes or no), and smoking status (yes or no). Women completed an additional questionnaire regarding menstrual history and menopausal status (pre- or post-menopausal) [17], [19].

### Statistical analysis

All data are reported as mean ± standard deviation (SD) unless otherwise stated. Participants with daily total caloric intake (kcal/day) falling outside the range of ±3SDs were considered outliers and excluded from further analyses to account for possible errors associated with over- or under-reporting of food intake on the FFQ. Insulin, HOMA-IR, and HOMA-β were log-transformed to normalize distributions and meet the assumptions of statistical tests. The sample size for the study was 2295 participants (590 men, 1705 women). Differences in physical, biochemical, and dietary patterns between men and women were assessed using one-way ANOVA. Subjects were subdivided by adiposity into Normal-Weight (NW), Overweight (OW), and Obese (OB) groups based on %BF measured by DXA according to the age and gender-specific criteria recommended by Bray [25]. Differences in physical, biochemical, and dietary patterns among adiposity groups was assessed using a one-way ANCOVA controlling for age, gender, total caloric intake, physical activity, medication use and smoking and menopause. As the number of underweight subjects was too low (n = 28) to perform effective statistical analysis, they were excluded from these analyses. To initially explore the relationship between dietary magnesium and IR (fasting glucose (mmol/l), insulin (pmol/l), HOMA-IR, HOMA-β), participants were divided into a tertile (low, medium, or high) based upon dietary magnesium intake (mg/day) which were assessed using an ANCOVA controlling for age, gender, total caloric intake, physical activity, medication use, smoking, menopause and %BF. Subsequently, multiple regression analyses were used to more rigorously explore the potential association of dietary magnesium intake (g/kg body weight) with insulin resistance (HOMA-IR) within the entire cohort and among men and women adjusting for age, gender, caloric intake, physical activity, medication use, smoking, menopause and %BF or BMI. However, only caloric intake, physical activity, medication use, menopause and %BF or BMI were statistically considered as confounding variables within the regression models. In addition, due to the significant interaction of adiposity and menopause on the association between dietary magnesium intake and insulin resistance, multiple regression analysis was performed among normal-weight, overweight and obese subjects along with pre-menopausal (n = 834) and post-menopausal (n = 577) women. Multiple regression analyses were performed on subjects stratified into tertiles (low, medium, or high) based upon both BMI and %BF measured from DXA to assess the difference in their adjustments on the relationship between dietary magnesium intake and insulin resistance. Lastly, Binary logistic analysis was also performed to explore the association between magnesium intake (g/kg/day) and diabetes status. Diabetes status was defined by a fasting glucose cutoff (glucose >7.0 mmol/L) together with those individuals who reported they were diabetics. Under the aforementioned definition, 102 subjects were labeled as diabetics for this analysis. Age, gender, caloric intake, physical activity, medication use, menopausal status, and smoking status were used as covariates. However, only caloric intake, medication status and age were statistically considered as confounding variables within the binary logistic model. Some subjects had missing data in one or more measurements. Five subjects did not disclose their smoking status and eight subjects did not respond to the medication use question. 1981 participants had all complete dataset for all variables measured to be used for the ANCOVA and multiple regression analysis. All statistical analyses were performed with and without diabetic subjects; however none of the results were not affected. All statistical analyses were performed using PASW 19.0 (SPSS Inc., Chicago, IL). All tests were two-sided and a p-value <0.05 was considered to be statistically significant.

## Results

### Physical, Biochemical, and Dietary Characteristics of Normal-weight, Overweight, and Obese participants

Physical, biochemical, and dietary characteristics for men and women along with those for normal-weight, overweight and obese participants are presented in **Table 1**. Glucose, insulin, and HOMA-IR were significantly greater for men than women, but HOMA-β was significantly greater among women. In terms of dietary intake, male participants had a significantly higher total daily caloric and magnesium intake than females. However, women had significantly greater magnesium intake (mg/day/kg) per kilogram body weight than men. Insulin, glucose, HOMA-β, and HOMA-IR values were greater among overweight and obese subjects, which we have previously described [17], [19], [20]. Individuals with the highest insulin, HOMA-IR and HOMA-β had the highest levels of adiposity. In addition, subjects with highest levels of adiposity had the lowest levels of magnesium intake (mg/day), and magnesium intake per kilogram of body weight (mg/day/kg). The findings remained significant after controlling for total age, gender, caloric intake, physical activity, medication use, menopausal status and smoking status. Serum magnesium was not significantly different among adiposity groups (**Table 1**).

**Table 1:** Physical, Biochemical, and Dietary Intake Characteristics of Normal-weight, Overweight, and Obese Participants.

		Gender		Percent Body Fat - Bray Criteria			
	Entire Cohort	Male	Female	Normal Weight	Overweight	Obese	p
	(n = 2295)	(n = 590)	(n = 1705)	(n = 547)	(n = 600)	(n = 781)	
Age (yr) ^3^	43.16±13	41.04±14.4	43.92±12.4	40.96±14.34	45.02±12.53	44.14±12.3	<0.001
Weight (kg)^2^	74.72±16.5	86.86±15.8	70.5±14.5	64.63±10.47	71.22±11.37	86.12±16.53	<0.001
Height (cm)^2^	165.8±8.6	176.09±6.5	162.24±5.9	166.68±8.5	165.32±8.2	165.98±9.0	NS
Waist (cm)^2^	93.07±15	98.68±13.9	91.13±14.9	82.35±9	90.8±10.3	103.68±14.5	<0.001
Hip (cm)^3^	101.66±11.9	100.8±10.4	101.94±12.4	92.76±6.6	99.48±7.2	110.25±11.8	<0.001
Waist-Hip Ratio^2^	0.91±0.1	0.98±0.1	0.89±0.1	0.89±0.1	0.91±0.1	0.93±0.1	<0.001
BMI (kg/m^2^)^2^	27.1±5.2	27.98±4.6	26.79±5.4	23.17±2.6	25.97±2.9	31.19±5.0	<0.001
Total Body Fat (%)^3^	35.17±9.1	26.31±7.7	38.22±7.5	26.86±6.5	34.86±5.7	42.12±6.6	<0.001
Glucose (mmol/L)^2^	5.16±0.9	5.35±1	5.09±0.9	4.98±0.7	5.15±0.8	5.33±1.1	<0.001
Insulin (pmol/L)^2^	68.86±67.7	73.53±63	67.2±69.3	50.58±59.3	64.16±72.8	84.74±73.2	<0.001
HOMA-IR^2^	2.39±3.3	2.65±3.4	2.3±3.3	1.72±3.4	2.21±3.6	3.0±3.4	<0.001
HOMA-β^3^	133.99±165	127.24±129.4	136.25±175.8	111.98±176.3	122.75±102.65	153.38±204.4	<0.001
Magnesium intake (mg/day)^2^	368.57±210.4	394.29±228.7	359.66±203.3	415.96±253.78	354.06±171.3	351.5±208.7	0.003
Magnesium intake (mg/day/kg)^3^	5.15±3.1	4.68±2.8	5.3±3.26	6.51±3.9	5.05±2.5	4.2±2.4	<0.001
Serum Magnesium (mmol/L)^2^	0.88±0.1	0.9±0.09	0.87±0.08	0.88±0.08	0.88±0.08	0.87±0.08	NS
Calories (kcal/day)^2^	1985.8±878.6	2246.5±982.7	1896.1±821.7	2118.6±937.1	1885.9±764.62	1958±897.4	<0.001

1 Data presented as mean ± SD. Homeostasis model assessment of insulin resistance (HOMA-IR) and β-cell function (HOMA-β). Gender differences were assessed with a one-way ANOVA. Subjects were also stratified into normal-weight, overweight and obese based upon %BF according to the Bray criteria (25). Adiposity differences were assessed with an ANCOVA controlling for caloric intake, physical activity, medication use, and menopause. ^2^Significantly greater for men compared to women. ^3^Significantly greater for women compared to men. ^4^Statistical significance for one-way ANOVA and ANCOVA were set to p<0.05 (IBM SPSS Statistics 19).

### Insulin Resistance Among Low, Medium, and High Dietary Magnesium Intake Groups

Physical, biochemical, and dietary characteristics were assessed among a tertile (low, medium, or high) based upon dietary magnesium intake (mg/day) (**Table 2**). Those individuals with the highest magnesium intake had the lowest insulin, HOMA-IR and HOMA-ß. Individuals with the lowest magnesium intake had the highest fasting insulin levels, HOMA-IR, and HOMA-ß. The findings remained significant after accounting for age, gender, caloric intake, physical activity, medication use, smoking, menopause and %BF. The dietary magnesium intake ranged from; 33.06 to 270.83 mg/day in the low dietary magnesium intake group, 270.84 to 393.66 mg/day in the medium dietary magnesium intake group, and 394.07 to 2493.01 mg/day in the high dietary magnesium intake group. Dietary magnesium intake per kilogram of body weight ranged from; 0.43 to 5.3 mg/kg/day in the low dietary magnesium intake group, 2.0 to 8.18 mg/kg/day in the medium dietary magnesium intake group, and 3.17 to 44.84 mg/kg/day in the high dietary magnesium intake group.

**Table 2:** Physical, Biochemical, and Dietary Intake Characteristics According to Magnesium Intake.

	Dietary Magnesium Intake (mg/day)			
	Low Mg Intake	Medium Mg Intake	High Mg Intake	p
	(n = 765)	(n = 765)	(n = 765)	–
Age (yr)	43.78±12.1	43.49±12.3	42.22±14.5	–
Weight (kg)	74.63±16.7	74.46±15.9	75.04±16.8	NS
Height (cm)	164.7±8.2	165.65±8.3	167.07±9.0	NS
Waist (cm)	94.3±15.0	92.94±15.4	91.93±14.5	NS
Hip (cm)	102.99±11.8	101.78±11.9	100.18±11.9	NS
Waist-Hip Ratio	0.91±0.08	0.91±0.08	0.92±0.09	NS
BMI (kg/m^2^)	27.4±5.3	27.08±5.1	26.79±5.2	NS
Total Body Fat (%)	36.68±8.3	35.3±9.1	33.5±9.7	–
Glucose (mmol/L)	5.18±0.8	5.18±1.0	5.17±1.0	NS
Insulin (pmol/L)	72.82±68.6	71.45±91.3	60.57±42.9	<0.001
HOMA-IR	2.49±2.5	2.59±5.2	2.07±1.8	0.003
HOMA-β	142.37±213.3	135.7±182.0	116.24±89.9	<0.001
Magnesium intake (mg/day)	201.57±48.6	328.85±36.3	575.98±224.1	<0.001
Magnesium intake (mg/day/kg)	2.83±0.9	4.59±1.0	7.99±3.6	<0.001
Serum Magnesium (mmol/L)	0.88±0.08	0.88±0.07	0.89±0.08	0.023
Calories (kcal/day)	1299.11±409.9	1904.21±440.42	2747.22±928.49	–

1 Data presented as mean ± SD. Homeostasis model assessment of insulin resistance (HOMA-IR) and β-cell function (HOMA-β).

2 Subjects were stratified into a tertile (low, medium and high) based upon magnesium intake (mg/day).

3 Magnesium intake group differences were assessed with an ANCOVA controlling for caloric intake, physical activity, medication use, menopause and %BF.

4 Statistical significance for one-way ANCOVA was set to p<0.05 (IBM SPSS Statistics 19).

### Relationship between Dietary Magnesium and Insulin Resistance

Unadjusted and adjusted linear regression analysis results of dietary magnesium intake on insulin resistance are shown in **Table 3**. There was a significant negative association between dietary magnesium intake (g/day/kg) with HOMA-IR in the entire cohort before and after adjusting for caloric intake, physical activity, medication use, menopausal status and %BF or BMI. In addition, the negative association between magnesium intake and insulin resistance was greater having adjusted for adiposity (%BF) over adjusting for BMI (**Table 3**). Multiple regression analysis of the entire study cohort also revealed a significant interaction of adiposity (%BF & BMI) with the negative association of magnesium intake with insulin resistance. Therefore, multiple regression analysis was performed on NW, OW and OB groups based upon %BF according to the Bray Criteria (**Table 3**). Magnesium intake was found to be significantly negatively associated with HOMA-IR among all adiposity groups. This inverse relationship was greater among overweight and obese subjects and pre-menopausal women.

**Table 3:** Regression Models of Magnesium Intake on Insulin Resistance.

	Unadjusted			Adjusted			Adjusted+%BF			Adjusted+BMI		
	β	β*	p	β	β*	p	β	β*	p	β	β*	p
HOMA-IR												
Entire Cohort	−21.03 (1.8)	−0.232 (0.02)	<0.0001	−33.75 (2.9)	−0.374 (0.03)	<0.0001	−19.33 (2.9)	−0.214 (0.03)	<0.0001	−11.65 (2.9)	−0.129 (0.03)	<0.0001
Normal–weight	−7.68(2.6)	−0.085 (0.03)	0.003	−10.92 (4.0)	−0.120 (0.05)	<0.0001	–	**–**	**–**	**–**	**–**	**–**
Overweight	−12.84 (3.9)	−0.141 (0.04)	0.001	−30.13 (6.9)	−0.332 (0.08)	<0.0001	**–**	**–**	**–**	**–**	**–**	**–**
Obese	−23.79 (4.0)	−0.262 (0.04)	<0.0001	−45.97 (6.5)	−0.506 (0.07)	<0.0001	**–**	**–**	**–**	**–**	**–**	**–**
Pre-Menopause	−15.07 (2.5)	−0.188 (0.03)	<0.0001	−35.32 (4.4)	−0.439 (0.05)	<0.0001	−20.4(4.6)	−0.254 (0.05)	<0.001	−13.57 (4.4)	−0.169 (0.05)	0.002
Post-Menopause	−18.91 (3.5)	−0.208 (0.04)	<0.0001	−27.44 (4.6)	−0.311 (0.05)	<0.0001	−16.89 (4.8)	−0.191 (0.05)	<0.001	−7.32(4.6)	−0.083 (0.05)	NS

1 Regression model adjusted for caloric intake, physical activity, medication use and menopausal status.

2 β = Unstandardized Beta (standard error), β* = Standardized Beta (standard error), Magnesium intake (g/day/kg).

3 Normal-weight, overweight and obese groups are based upon %BF according to the Bray criteria (25).

4 Magnesium intake (Pre-Menopause 360.63±209.8 mg/day, Post-Menopause 353.82±192.9 mg/day) (Entire cohort, Normal-weight, Overweight, & Obese – See Table.1).

5 Statistical significance was set to p<0.05 (IBM SPSS Statistics 19).

### Relationship between Dietary Magnesium and Insulin Resistance Among Low, Medium and High %BF and BMI

Unadjusted and adjusted linear regression analysis results of dietary magnesium intake (g/day/kg) on insulin resistance among low, medium and high BMI and %BF groups are shown in **Table 4**. Magnesium intake was increasingly more negatively associated with HOMA-IR the greater the adiposity (%BF) or body mass index (BMI). Moreover, the negative association between dietary magnesium intake and insulin resistance was more prevalent according to a %BF classification than a BMI classification (**Table 4**).

**Table 4:** Regression Models of Magnesium Intake on Insulin Resistance based upon %BF and BMI.

	Body Fat Percentage													
	Low			Medium					High					
	β	β*	p	β	β*		p		β		β*		p	
Entire Cohort														
Unadjusted	−14.98 (2.5)	−0.165 (0.03)	<0.0001	−17.36 (3.4)	−0.191 (0.04)		<0.0001		−20.98 (4.1)		−0.231 (0.05)		<0.0001	
Adjusted	−14.30 (4.1)	−0.157 (0.04)	0.001	−23.39 (6.0)	−0.258 (0.07)		0.0002		−45.59 (6.48)		−0.502 (0.07)		0.000	
	**Body Mass Index**													
Entire Cohort														
Unadjusted	−3.89 (2.1)	−0.043 (0.02)	NS	−12.4 (3.5)		−0.137 (0.04)		0.0004		−24.27 (4.7)		−0.267 (0.05)		<0.0001
Adjusted	−9.97 (3.5)	−0.110 (0.04)	0.004	−12.8 (6.4)		−0.141 (0.07)		0.047		−57.6 (7.9)		−0.446 (0.08)		0.000

1 Regression model adjusted for caloric intake, physical activity, medication use and menopausal status.Subjects were also stratified into a tertiles(Low, Medium and High) based upon %BF and BMI.

2 β = Unstandardized Beta (standard error), β* = Standardized Beta (standard error), Magnesium intake (g/day/kg).

3 Magnesium intake (Low BMI 409.78±243.5 mg/day, Medium BMI 353.24±180.9 mg/day, High BMI 342.76±196.1 mg/day) (Low %BF 387.5±230.3 mg/day, Medium %BF 360.54±187.5 mg/day, High %BF 357.68±210.7 mg/day).

4 Statistical significance was set to p<0.05 (IBM SPSS Statistics 19).

### Relationship between Dietary Magnesium and Diabetes Status

Binary logistic analysis, performed to explore the association between magnesium intake (g/kg/day) with diabetes status, revealed that magnesium intake (g/kg/day) was significantly negatively associated with diabetes status (n = 102, Unstandardized β = −499.80, standard error 79.45, p<0.0001). Caloric intake, medication status and age were statistically considered as confounding variables within the binary logistic model.

### Serum Magnesium and Markers of Insulin Resistance

The relationship between serum magnesium and insulin resistance was also explored. Although serum magnesium concentration concomitantly increased with dietary magnesium intake, it was not found to be significantly associated with insulin resistance in the Newfoundland population (data not shown).

## Discussion

The noteworthy finding of the present investigation was a beneficial dose dependent relationship between dietary magnesium intake and insulin resistance, independent of age gender, total caloric intake, physical activity, medication use, menopause, and adiposity. However, this favorable association was more significant in overweight and obese subjects suggesting that this population may be more sensitive to the beneficial effects of dietary magnesium intake. Obesity, as a disorder, is a well-known condition which can place individuals at a significantly elevated risk for impaired insulin action [26] and various metabolic abnormalities such as hypertension, dyslipidemia, and a reduction in glucose tolerance [26]. The functional relationship between various hormones and obesity-related conditions is a significant focus of obesity research, however diet composition has become increasingly recognized and studied [2], [4]. One study specifically demonstrated that an increase in dietary magnesium can significantly improve insulin sensitivity [27]. Magnesium has been proposed to be functionally related to glucose metabolism through an interaction with tyrosine-kinase activity on the insulin receptor which is associated with the development of insulin resistance and type 2 diabetes [28]. Studies have suggested that the effect of dietary magnesium on decreasing markers of IR [11], [12] and development of type 2 diabetes is more pronounced in overweight patients [11], [12]. However, body fat percentage was not measured in these studies. This is an especially important point to consider when the relative amount and distribution of adipose tissue, both important determinants of insulin sensitivity, cannot be determined by the body mass index [29]–[31].

In fact the majority of studies evaluating the association between dietary magnesium and insulin resistance have only utilized BMI when attempting to control for adiposity, bearing in mind that percent body fat and BMI likely represent different physiological entities. Having adjusted for the influence of %BF and BMI on the association of dietary magnesium intake with insulin resistance, we observed that this inverse association was stronger after having adjusted for %BF than for BMI which further supports our hypothesis. Considering that our laboratory and others have revealed that BMI is not an accurate measure of body fat due to its inability to differentiate fat mass from fat free mass [20], [32], [33], it is implicit that BMI cannot represent adiposity. We found that overweight and obese individuals, defined by a high resolution adiposity measurement, are more sensitive to the beneficial effect of magnesium intake on insulin resistance. Our current findings, taken together with others of others [11], [12] provides strong evidence that overweight individuals, classified by either %BF or BMI, could potentially benefit from an increase in magnesium intake. It is possible that overweight and obese individuals are better able to absorb and metabolize magnesium, thereby enhancing its action at the insulin receptor and promoting insulin sensitivity. To our knowledge, this study is the first large cross-sectional study to systematically control for major confounding factors including %BF when analyzing the relationship between dietary magnesium intake and markers of insulin resistance. This study is also the first investigation to observe that dietary magnesium is more strongly associated with insulin resistance when adjusting for %BF than BMI.

The association between dietary magnesium intake and insulin resistance was examined with regards smoking [34], menopause [12] and medication [35], [36] as possible confounding factors for this relationship. Evidence suggests that nicotine intake may increase insulin resistance, however we did not find a significant association of smoking status with HOMA-IR or magnesium intake. In addition, during the regression model development smoking status failed to reach statistical significance and was not included in the regression model. Our data would suggest that smoking is not a critical confounding factor regarding the beneficial effect of dietary magnesium intake on insulin resistance in the Newfoundland population. However, this finding may be due to the significantly smaller sample size of smokers (n = 222) to non-smokers (n = 1754) in our study cohort. Further study is needed to elucidate the potential interaction of smoking on the relationship of dietary magnesium intake and insulin resistance. Medication status was significantly associated with insulin resistance and there is evidence to suggest that various medications inhibit magnesium re-absorption in the kidney which can result in magnesium deficiency [35], [36]. However, we were unable to find a significant interaction of medication use on the inverse association with magnesium intake and insulin resistance. Pre-menopausal women were more significantly associated the beneficial effects of magnesium intake on insulin resistance than post-menopausal women. This finding is strengthened by the Shanghai Women’s Health Study, designed to assess the prospective risk of type 2 diabetes, which found a statistically significant negative correlation between magnesium intake and type 2 diabetes risk in pre-menopausal women only [12]. We considered, since over 68% of our post-menopausal women are medication users, that the lack of association of magnesium intake with insulin resistance could be drug related interference. However, this relationship remained absent among post-menopausal women whether or not medication status was included in the regression model. Therefore, our data would suggest that the beneficial effects of magnesium intake are less sensitive among post-menopausal women and further study is needed to explore the physiological mechanism involved. Lastly, we discovered that magnesium intake was significantly negatively associated with diabetes. Considering that insulin resistance is a significant clinical symptom of diabetes and that insulin resistance was significantly inversely associated with magnesium intake this, finding was not surprising. Our results suggest that the potential beneficial influence of magnesium intake exists in a wide range of populations from the general population to insulin resistance and to those people struggling with struggling with diabetes in Newfoundland and Labrador.

Aside from investigating the influence of %BF and BMI on the association between magnesium intake and insulin resistance in the general population and the influence that an adiposity status defined by %BF has on this relationship, we also investigated whether an adiposity status defined by BMI would have a different effect than %BF on this association.

Considering that the adiposity status criteria for the World Health Organization (WHO) [1] is considerably different from that developed by Bray et al. [25], the association between dietary magnesium intake and insulin resistance was examined among low, medium and high tertiles according to %BF and BMI. Our data revealed that the inverse relationship between dietary magnesium intake and insulin resistance was progressively stronger the greater the %BF or BMI. However, the aforementioned association was more significantly pronounced for concomitant increases in %BF over BMI. Consider that %BF is a more direct measure of adiposity than BMI, together with our current findings, we recommend that %BF be used when considering adiposity as a factor.

The apparent protective role of magnesium on IR and type 2 diabetes has not been fully explained but is likely due to enhanced insulin sensitivity through multiple mechanisms. For example, phosphorylation of the tyrosine kinase enzyme of the insulin receptor, required for post-receptor insulin sensitivity and subsequent insulin-mediated glucose uptake, is dependent on adequate intracellular concentrations of magnesium [37]. As such, we also chose to investigate the relationship between serum magnesium and markers of IR; no significant association between them was observed. Inconsistencies have been present in previous studies examining this relationship, some supporting a negative correlation [38], [39] and others not [10]. One possible explanation for these differences is that serum magnesium may not accurately reflect intracellular magnesium levels, which may be low, even when serum levels are within the normal range [14]. Additional studies are warranted to further clarify the relationship between serum magnesium and markers of IR.

Our study had certain limitations, many of which were due to its cross-sectional design. Firstly, our use of a FFQ to evaluate patterns of dietary intake raises the possibility of recall bias by subjects. However, the Willett FFQ chosen for this study is one of the most commonly applied tool for the evaluation of dietary intake in epidemiologic population-based studies [22], [23]. In addition, dietary magnesium is highly correlated with other micronutrients and dietary components believed to affect insulin sensitivity, such as vegetables, fruits, potassium, calcium, and fiber. Thus, it is very difficult to separate their independent effects [7], [12]. In addition, to avoid over-adjustment, we opted not to control for every available nutrient in our analysis. We did not measure or account for magnesium supplementation regarding daily magnesium intake, which could have potentially reduced the strength of the inverse association found between magnesium intake and IR makers. Furthermore, the reliability of serum magnesium levels in recognizing total body magnesium deficiency is unclear. Although intracellular magnesium concentrations are believed to provide a more accurate estimation of magnesium status, they are not generally easily measured [38]. Finally, our study enrolled Caucasian Newfoundlanders, so our findings may not be applicable to those from other ethnicities [16], [40].

In summary, our cross-sectional study investigated the relationship between dietary magnesium intake and insulin resistance among 2295 Newfoundlanders and Labradoreans. To our knowledge this study is the most comprehensive of its kind having controlled for major confounding factors, most specifically being dual energy x-ray absorptiometry (DXA) determined body fat percentage. Our findings suggest that higher dietary magnesium intake is associated with improved insulin sensitivity and this effect is particularly beneficial for overweight and obese individuals in the general population along with pre-menopausal women.

We also provide the first evidence that the association between dietary magnesium and insulin resistance is more strongly associated with %BF than BMI and the concomitant increase in %BF over BMI. Due to the fact that %BF more accurately represents adiposity than BMI, caution should be taken when attempting to utilize BMI as a measure of adiposity. Further large-scale prospective studies, where body fat is adequately accounted for and which enroll various ethnic groups, are needed to further elucidate the role of dietary magnesium in improving insulin function and preventing diabetes.
